# Evaluation of secondhand smoke effects on CFTR function in vivo

**DOI:** 10.1186/s12931-020-1324-3

**Published:** 2020-03-20

**Authors:** Lawrence W. Rasmussen, Denise Stanford, Krina Patel, S. Vamsee Raju

**Affiliations:** 1 Departments of Medicine, The University of Alabama at Birmingham, Birmingham, AL USA; 2 Environmental Health Sciences, The University of Alabama at Birmingham, Birmingham, AL USA; 3 Gregory Fleming James Cystic Fibrosis Research Center, The University of Alabama at Birmingham, Birmingham, AL USA; 4 Cell, Developmental, and Integrative Biology, The University of Alabama at Birmingham, Birmingham, AL USA

## Background

Cystic fibrosis transmembrane conductance regulator (CFTR) is an anion channel expressed on the mucosal surface of epithelial cells lining several tissues including airways. Inherited defects in the CFTR gene cause cystic fibrosis (CF), an autosomal recessive disease causing a progressive decline in lung function ultimately resulting in reduced life span. CF patients exhibit impaired mucociliary clearance (MCC), a principle defense of airways against inhaled irritants and pathogens. The MCC apparatus consists of airway surface liquid (ASL), which has a mucus layer that traps pathogens to be removed by the coordinated beating of surface cilia [1]. Physiologic levels of ASL and PCL determine the effectiveness of MCC, a process tightly regulated through CFTR activity [2, 3]. As a result of reduced CFTR-mediated ion transport, CF airways fail to maintain optimal surface hydration and clear inhaled pathogens. Decreased MCC efficiency causes accumulation of thick mucus, opportunistic infections, and chronic inflammation. Beyond inherited defects, several different environmental insults can also negatively affect CFTR function even in those with normal genetics. For example, exposure to cigarette smoke is known to cause decreased CFTR activity in mice [4], rats [5], ferrets [6], dogs [7], and humans [8, 9]. Reports also suggest excessive alcohol intake [10] and exposure to cadmium [11] and arsenic [12, 13] result in diminished CFTR activity supporting the concept of ‘acquired CFTR dysfunction’.

Secondhand smoke (SHS) exposure is an important health concern for many never smokers [14]. In spite of regulations limiting smoking in many public places, there are 1.8 billion non-smokers around the world that are frequently exposed to SHS resulting in more than 600,000 deaths per year [15, 16]. Exposure to SHS is a risk factor for respiratory infections and pulmonary diseases including lung cancer, asthma, and chronic obstructive pulmonary disease (COPD), the third leading cause of death in the U. S [17–20]. However, there is currently a limited understanding of how SHS exposure alters lung physiology to cause these diseases.

Savitski et al. reported that SHS exposure, like mainstream smoking, results in decreased CFTR anion transport in human bronchial epithelial cells [21], suggesting CFTR abnormality may explain delayed mucociliary clearance and increased disease burden in never smokers [22, 23]. However, the observation of SHS-induced defects in CFTR is yet to be verified in a suitable animal model. Moreover, the functional consequences of such CFTR dysfunction on mucus physiology remain unexplored. To address these important follow-up questions, we have monitored CFTR-mediated ion transport in A/J mice exposed to SHS. We have previously found that A/J mice exhibit susceptibility to smoking induced changes in CFTR function in a dose-dependent manner [4]. Based on these data, we have used A/J mice as an in vivo model to test the hypothesis that *SHS reduces CFTR function and disrupts physiologically optimal airway surface hydration and mucociliary transport.*

## Methods

### Secondhand smoke exposures

#### In vivo

Murine protocols were reviewed and approved by the University of Alabama at Birmingham Institutional Animal Care and Use Committee. “Gender matched 8-week-old A/J mice were restrained in custom-designed tubes (4–5 in. long with an inside diameter of 1 in.) and attached to nose-only inhalation tower connected to a supply of sidestream cigarette smoke from 3R4F research cigarettes (University of Kentucky, Lexington, KY) generated by a computer-controlled cigarette smoke generator (In Expose, Scireq, Montreal, Canada). The exhaust line at the end of the plenum was connected to a vacuum source at a negative pressure of 3 L/min for the continued flow of smoke [4, 24]. Using this setup, we achieved a steady flow of SHS at a particulate concentration of 0.09 mg/L of total particulate matter”.

#### In vitro

Use of Primary human bronchial epithelial (HBE) cells from non-CF CFTR+/+ subjects was approved by the Institutional Review Boards at the University of Alabama Birmingham. HBE cells were generated from lung explants by previously described procedures [25]. Both primary HBE cells and immortalized 16-HBE cells were expanded in submerged cultures and transferred to Transwell filter inserts for culturing at air-liquid interphase until cells were terminally differentiated into ciliated epithelium. Cells were placed in a custom-built chamber and exposed for 10 min to side stream smoke (0.09 mg/L of total particulate matter) generated by 3R4F cigarettes from a cigarette smoking robot (CSR) as previously described [4]. The 10 min SHS exposure was previously found to be optimal for human bronchial epithelial cultures without causing nonspecific cell injury [21]. Control cells were exposed to matched room air for a similar duration.

### N-acetylcysteine (NAC) treatment

#### In vivo

Mice received drinking water supplemented with 40 mM NAC throughout the 5-week of exposure studies [26]. Water supplemented with NAC was adjusted to pH 7.4 with sodium hydroxide and replaced every 72 h. 40 mM NAC administered in this manner is known to achieve up to1 gram of NAC per kg of body weight each day in mice [27]. No differences in the amount of water consumed or, gain of body weight were observed between mice receiving NAC supplemented water and those receiving control water.

#### In vitro

HBE cells were treated with 300 μM of NAC (Sigma) in basolateral media added 30 min before SHS exposure. After SHS/control air exposure, cells were incubated for 1 h with NAC at 37 °C with 5% CO_2_ prior to CFTR assay [4].

### CFTR mediated anion transport assay

CFTR function in HBE cells and murine tracheal explants was estimated in short-circuit current (Isc) units with modified Ussing chambers (Physiologic Instruments, San Diego, CA) under voltage clamp conditions as previously reported [4, 28]. Both HBE cells and tissues were bathed in identical Ringers solution bubbled with 95% O_2_: 5% CO_2_ before sequential addition of apical amiloride (100 μM), apical low chloride Ringers, apical and basal forskolin (20 μM), and apical CFTR-inh172 (10 μM) or basal bumetanide (10 μM). Data was acquired until establishment of steady Isc plateaus or at least 5 min after each reagent addition in Ussing chambers. Changes in epithelial ion transport following the addition of each reagent were calculated in delta Isc per unit area of epithelial surface.

### In vivo CFTR assay by nasal potential difference (NPD) measurement

In vivo CFTR activity was assessed in murine nasal epithelium by NPD measurements [29]. Nasal catheter made of PE10 tubing was inserted into a single naris of anesthetized mice for sequential infusion of Ringers solution (baseline); Ringers + Amiloride (100 μM); Chloride-free Ringers solution consisting of KHPO (2.4 mM), KHPO (0.4 mM), Na Gluconate (115 mM), NaHCO (25 mM), and Ca Gluconate (1.24 mM); Chloride-free Ringers + forskolin (20 μM); and Chloride-free Ringers with 10 μM of CFTR-inh172. CFTR function was expressed in mean changes in voltage.

### μOCT image analysis

Freshly excised murine tracheae were imaged with a high-resolution reflectance imaging modality known as micro optical coherence tomography (μOCT), as previously described [30, 31]. Briefly, surface images from 8 different locations were acquired with an optical beam (Photonics Superk Extreme high power supercontinuum White Light Laser, NKT Photonics) scanning longitudinally along the tracheal ventral surface with the larynx end serving as a reference. Airway surface liquid (ASL) and periciliary liquid (PCL) depths were measured by ImageJ geometric tools. Mucociliary transport (MCT) rate was evaluated by tracking naturally occurring mucus particles traveling along the ventral surface over multiple frames and then converting pixel to micron through the initial image calibrations [32].

### Acrolein estimation by mass spectrometry

Cell-free PBS samples were exposed to room air or SHS under same conditions as that of HBE cell exposure with and without 300 μM of NAC, as performed elsewhere [33]. Immediately after exposure, PBS samples were processed to generate stable derivatives of acrolein for subsequent estimation by mass spectroscopy. PBS samples were loaded into the 10 kDa MWCO Amicon ultrafilters (Millipore, Billerica, MA) and centrifuged at high speed for 30 min at 4 °C. The filtrate was then derivatized using freshly prepared 2,4-diphenylhydrazine (DNP, Sigma, St Louis, MO) in acetonitrile/formic acid and allowed to react at room temperature for 1 hour. The solution was clarified by centrifugation at high speed for 5 min. Mass spectroscopy was conducted on an API-4000 (AB Sciex, Framingham, MA) equipped with a C18, 100A, 100 × 2.10 Kinetix column (Phenomenex, Torrance, CA). Standard solutions were prepared from acrolein-DNP solid synthesized in-house with > 95% purity by NMR. A gradient of 5–100% solutions containing acetonitrile and 10 mM ammonium acetate) was infused over 10 min at a flow rate of 0.3 ml/min. Analytes were modified with chemical ionization in negative mode. The 235–163 and 235–65 peaks were quantified relative to acrolein standards and the values averaged for each sample, as reported earlier [4].

### Statistics

Descriptive statistics were compared using Student’s t-test. Error bars represent SD unless noted in legend as SEM. All statistical tests were two-sided and were performed at 95% significance level (i.e., α = 0.05) using GraphPad Prism (La Jolla, CA). Statistical correlation between CFTR assay and various aspects of mucociliary apparatus (ASL depth, PCL depth, and MCT rate) were performed using SPSS ver. 25 (IBM, Armonk, NY); Pearson correlation matrix was performed with a two- tail test with confidence intervals set at 95%. All assumptions for using Pearson instead of Spearman correlation were verified before running correlation analyses. Finally, scatterplots representing relationships between dependent and independent variables were made in GraphPad Prism after confirming the validity of Pearson correlation matrix analysis by SPSS.

## Results

### Effect of secondhand smoke on CFTR function in vitro

**Fig. 1:**
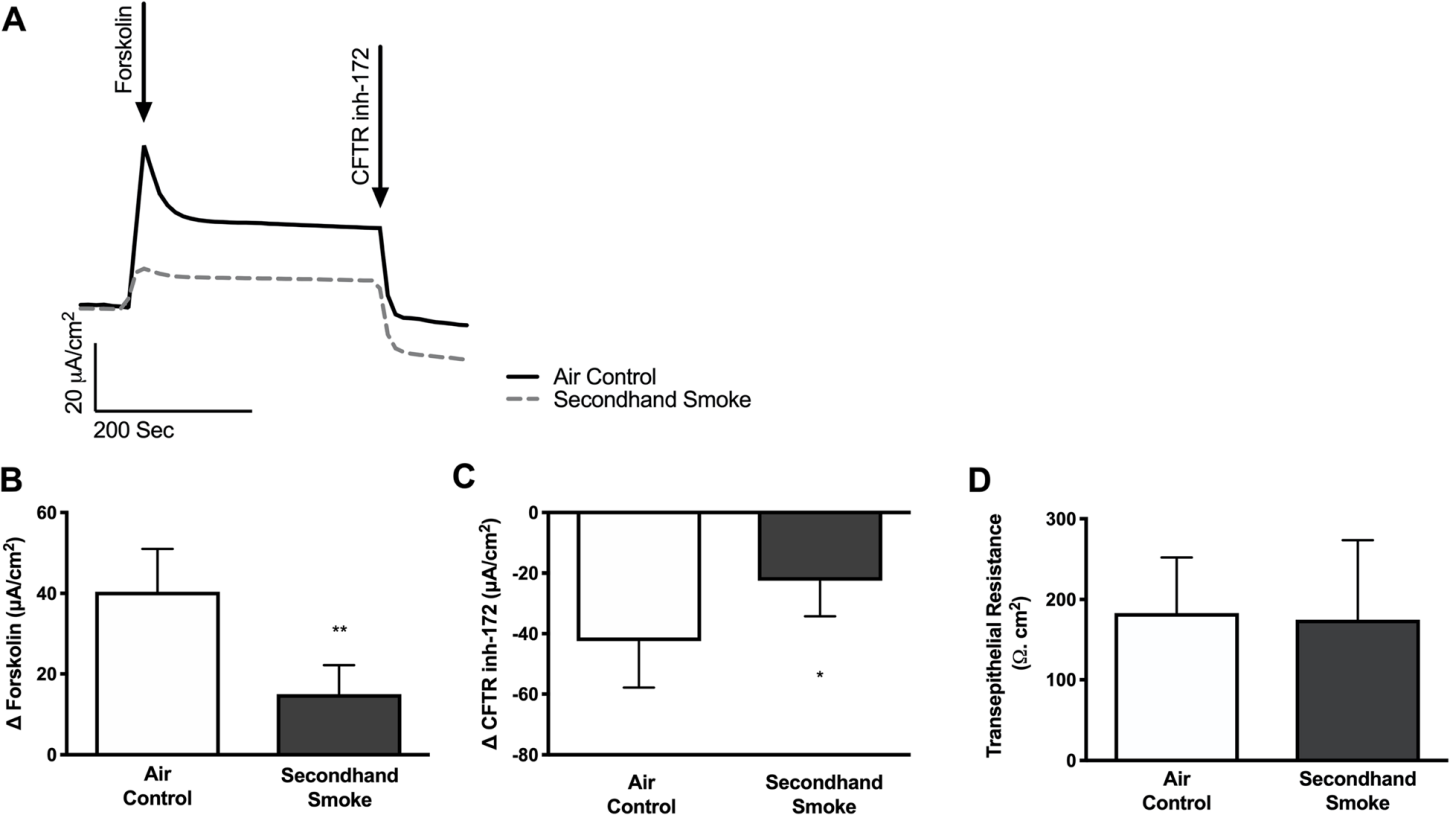
Effect of secondhand smoke on CFTR function in vitro. Primary human bronchial epithelial (HBE) cells were isolated from healthy non-smokers expressing wild type CFTR (CFTR +/+) and cultured at air liquid interface (ALI) until terminally differentiated into ciliated epithelium. HBE cell monolayers were exposed to secondhand smoke (SHS) for 10 min and CFTR function was measured in Ussing chambers under voltage clamp conditions. **a.** Representative tracings of forskolin stimulated CFTR ion transport in HBE cells. Addition of forskolin (20 μM) and CFTR_inh_172 (10 μM) are indicated. Summary of changes in CFTR-dependent anion transport are plotted for forskolin stimulation (**b**) and inactivation by specific inhibitor, CFTR_inh_172 (**c**). **d** Bar graph depicting transepithelial resistance in HBE cell monolayers after exposure to 10 min of air or SHS. *n* = 6, *P < 0.05, **P < 0.005

Since cigarette smoke is known to cause reduced CFTR mediated anion transport in both smokers as well as patients with COPD [14–17, 19], we hypothesized that acquired CFTR dysfunction may also be caused by exposure to SHS. To test his hypothesis, we exposed primary HBE cells expressing wild-type CFTR to side stream smoke and compared data to matched cells exposed to room air. SHS caused a pronounced loss of forskolin-stimulated CFTR activity (Δ forskolin Isc, air control 40.45 ± 4.3 μA/cm^2^, SHS 14.97 ± 2.9 μA/cm^2^, *p* < 0.005), consistent with prior studies with whole cigarette smoke [17, 19, 30, 34, 35] and cigarette smoke extract (CSE) [14, 36] (Fig. 1a, b). SHS also caused a significant reduction in transepithelial currents that are sensitive to CFTR inhibitor in these cells (Δ CFTRinh-172 Isc, air control − 42.4 ± 6.3 μA/cm^2^, SHS -22.42 ± 4.8 μA/cm^2^, *p* < 0.05), confirming the harmful effects of environmental exposure to SHS on CFTR despite normal expression at baseline (Fig. 1c). Moreover, when we assessed the viability of HBE cell monolayers by measuring transepithelial electrical resistance there were no differences between those exposed to SHS and controls indicating reduced CFTR activity was not due to toxicity but, likely outcome of specific effects of SHS on airway epithelium in vivo (Fig. 1d). These data are consistent with previous reports that indicate SHS exposure suppresses chloride ion transport function of CFTR in HBE cells in a dose-dependent manner [21].

### Secondhand smoke (SHS) exposure reduces CFTR function in a/J mice

**Fig. 2:**
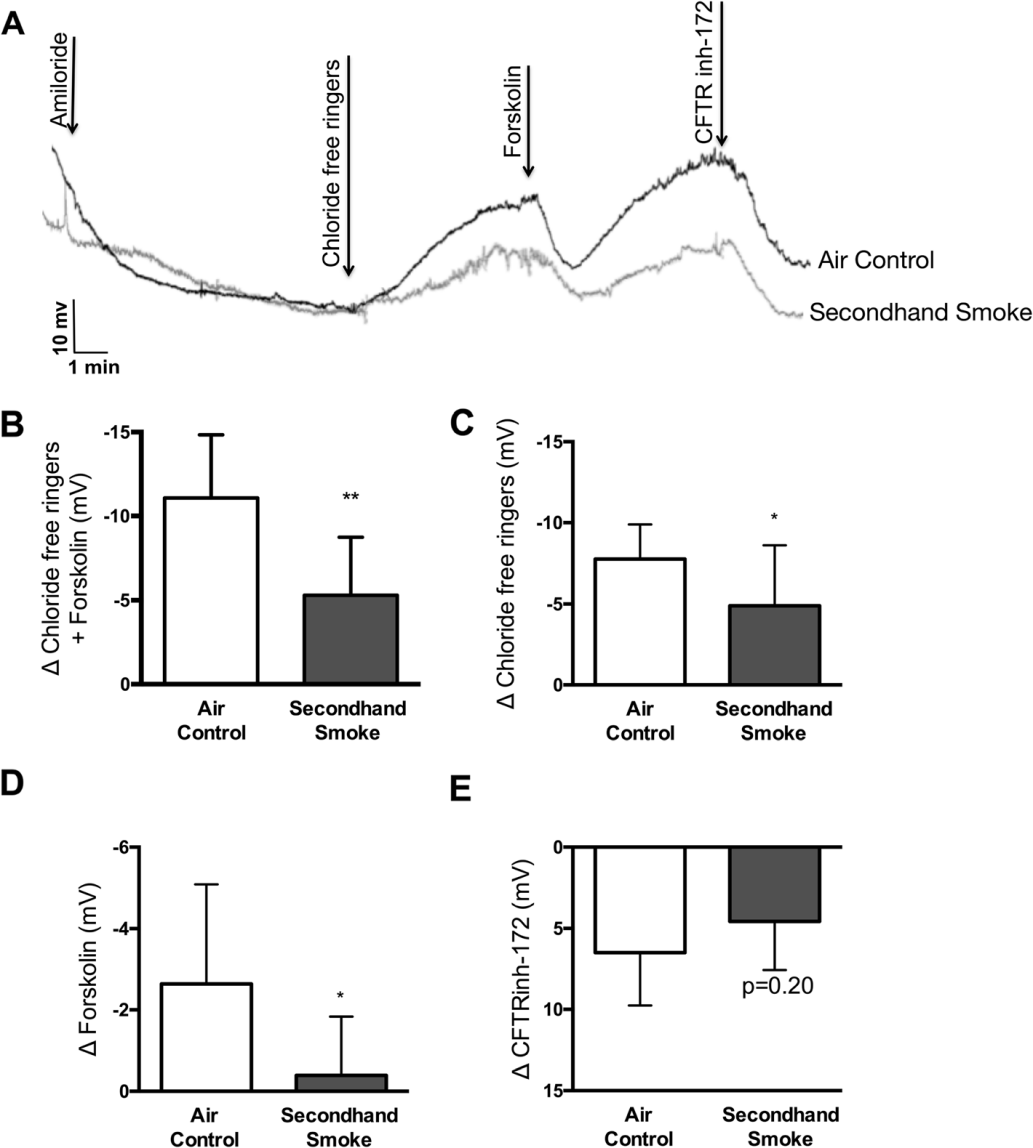
Secondhand smoke (SHS) exposure decreases CFTR-mediated chloride ion transport in vivo. **a.** Representative nasal potential difference (NPD) tracings acquired in mice exposed to control room air as a control or SHS for 6 weeks using a custom-built nose-only inhalation exposure system. **b**. Summary graph illustrates changes in total CFTR-dependent voltage following sequential infusion of chloride-free ringers and forskolin (10 μM). Reductions in CFTR activity are shown for the proportion of CFTR channels that were open at baseline and responded to chloride-free ringers alone (**c**) and separately for those that were closed until activation by forskolin (**d**). **e**. Differences in CFTR activity are represented following addition of CFTR-specific inhibitor CFTR_inh_172. *n* = 6–8, *P < 0.05; **P < 0.005

To verify in vitro findings regarding SHS effects in vivo*,* we exposed wildtype (*CFTR +/+*) A/J mice to side stream cigarette smoke generated from 3R4F research cigarettes for 5 weeks. To assess changes in CFTR activity, transepithelial ion transport was measured by nasal potential difference (NPD) under conscious sedation 12 h after the last episode of SHS exposure to minimize the influence of acute smoke effects (Fig. 2a). NPD is a reliable method for estimating in vivo CFTR function in laboratory animal models and is routinely used for CF diagnosis in the clinic. Compared to air exposed controls, mice exposed to SHS exhibited a 52% decrease (Δ chloride-free ringers + forskolin: SHS -5.30 ± 3.4 mV vs air control − 11.07 ± 3.7 mV, *p* < 0.005) in total CFTR function (Fig. 2b). More detailed analysis indicates, CFTR channels in open configuration (as determined by response to ion gradient set by the infusion of chloride-free Ringers) were reduced by 37% (Δ chloride-free ringers: SHS -4.89 ± 3.72 mV vs air control − 7.75 ± 2.14 mV, *p* < 0.05, Fig. 2c); whereas, channels that were in closed configuration (as determined by lack of response to chloride ion gradient, but increase in voltage following activation with forskolin) were reduced by 85% (Δ forskolin: SHS -0.39 ± 1.44 mV vs air control − 2.64 ± 2.45 mV, *p* < 0.05, Fig. 1d). These trends in CFTR activity were also reflected in voltage changes in response to CFTR-specific inhibitor, CFTR-inh172, in both groups of mice. While CFTR-inh172-mediated voltage was decreased by 30% in SHS-exposed mice (SHS 6.50 ± 3.27 mV vs air control 4.57 ± 3.0 mV, *p* = 0.20, Fig. 2e) the difference was not statistically significant suggesting partial efficacy of the inhibitor in vivo, consistent with previous observation across different species including, mice [34, 35].

**Fig. 3:**
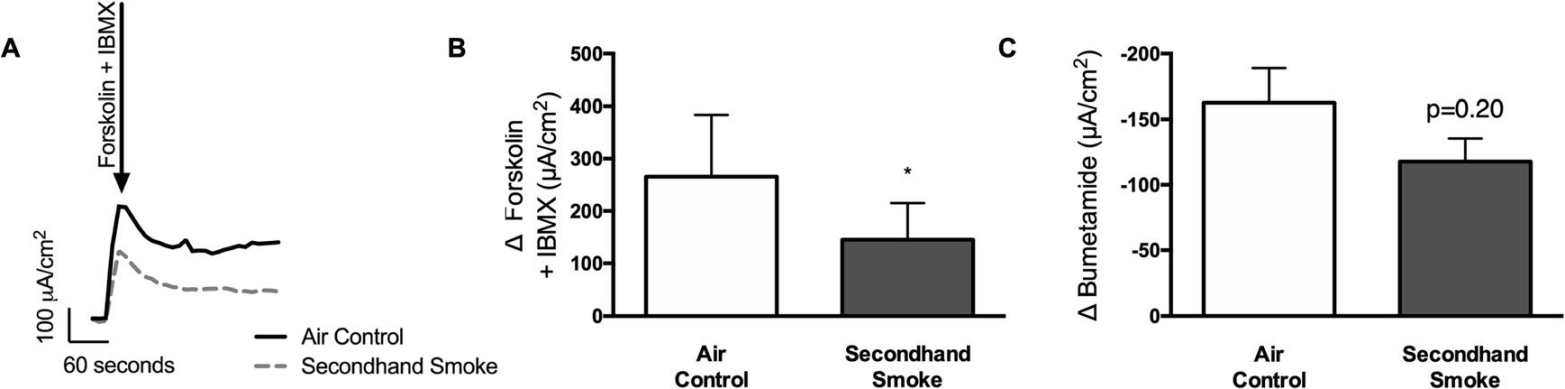
Secondhand smoke (SHS) decreases CFTR-mediated chloride ion transport in murine airways. **a**. Representative electrophysiology tracing of excised trachea from mice exposed to either control room air or SHS for 6 weeks and mounted in modified Ussing Chambers. **b**. Graph illustrates summary of CFTR-mediated short circuit currents (Isc) in response to stimulation with forskolin (10 μM) and IBMX (100 μM) under the conditions of basal-apical Cl^−^ ion gradient and inhibited Na^+^ absorption by epithelial sodium channel (ENaC) by amiloride (100 μM). **c**. Graph demonstrating bumetanide (10uM) inhibition of basolateral NKCC1, a Na^+^/K^+^/2Cl^−^ cotransporter, confirmed reduction in CFTR-dependent chloride ion transport. n = 6–8, *P < 0.05; **P < 0.005

In addition to ion transport measurements across nasal epithelium, CFTR activity in airway epithelium was estimated by short circuit current (Isc) measurement in freshly excised trachea by Ussing chamber electrophysiology. In tracheal explants, CFTR-dependent Isc was estimated in response to a combination of cAMP agonists, forskolin and IBMX (a non-selective phosphodiesterase inhibitor), following inhibition of sodium ion absorption via ENaC channels with amiloride and basolateral-apical chloride ion gradient. As shown in Fig. 3a-b, SHS exposure lowers CFTR activity in murine trachea by 45% (SHS 145.4 ± 69.85 μA/cm^2^ vs air control 265.40 ± 118.10 μA/cm^2^, *p* < 0.05), which is consistent with in vivo NPD measurements shown in Fig. 2. In these murine tracheae, addition of CFTR-specific inhibitors CFTRinh-172 and GlyH101 resulted in no quantifiable change in Isc (data not shown). As a result, we evaluated chloride ion transport as a function of Isc change in response to bumetanide that blocks basolateral NKCC1, a Na^+^/K^+^/2Cl^−^ cotransporter that mainly generates chloride ion supply for CFTR channels on the apical side [36]. Changes in Isc by bumetanide have been shown to reliably reflect chloride transport via apical CFTR channels in primary lung epithelial cells from human donors and rodent models (Fig. 3c) [37]. In SHS-exposed mice, bumetanide-sensitive tracheal Isc was suppressed by 28% when compared to control mice (SHS -117.70 ± 52.89 vs air control − 162.80 ± 82.48 μA/cm^2^ μA/cm^2^, *p* = 0.20). Taken together, these data establish that SHS exposure causes a sustained decrease in CFTR function in vivo*.*

### Acrolein mediates SHS-induced CFTR dysfunction

**Fig. 4:**
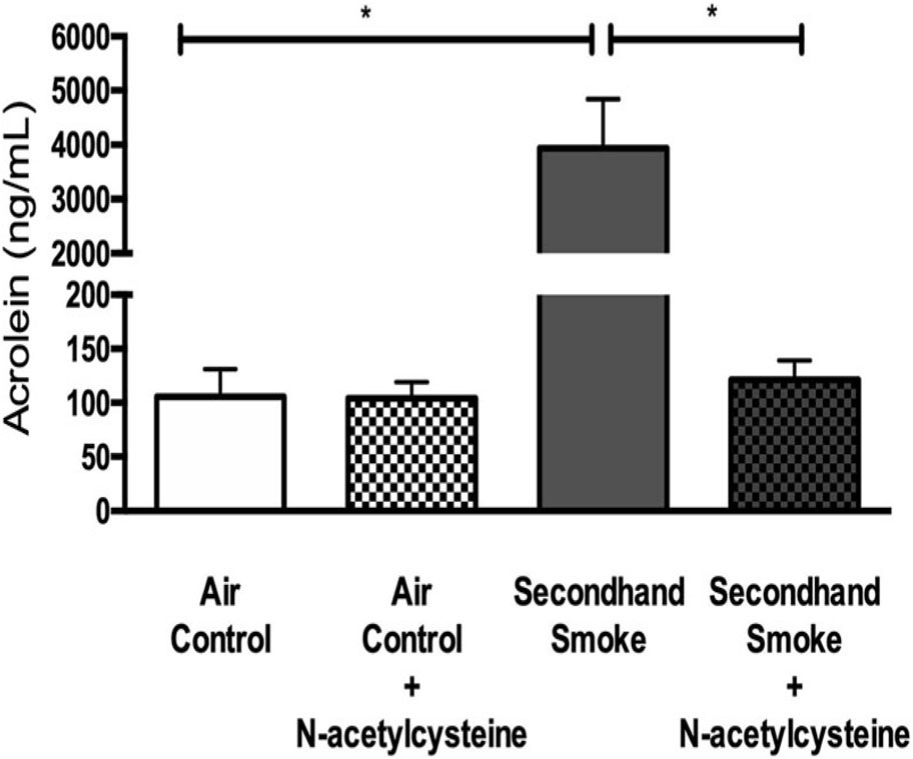
Acrolein, a highly reactive aldehyde known to inhibit CFTR function, is present in secondhand smoke (SHS) and is neutralized by N-acetylcysteine (NAC). Acrolein content was estimated by exposing cell-free PBS to SHS for 10 min under conditions to which bronchial epithelial cells and mice were exposed. Exposure samples were immediately derivatized with dinitrophenylhydrazine to generate stable acrolein intermediates for mass spectrometry chemical analysis. Summary of acrolein measured in presence and absence of NAC (300 μM) added to the sample collection buffer is shown. *n* = 4/condition, *P < 0.05

After establishing the biologic effects of SHS on CFTR function in vivo, we attempted to identify the key toxic agents present in SHS that may be responsible for these adverse effects on epithelial ion transport. Towards this objective, we first analyzed the presence of acrolein, a 3-carbon reactive aldehyde, in SHS. This is based on our previous findings that circulatory levels of acrolein are higher in smokers and acrolein can directly modify specific residues in CFTR protein to impact ion transport function [4, 38]. Initially, we attempted to estimate circulatory levels of free acrolein in murine serum samples using the same protocol that was successfully used for evaluation of human samples [4]. However, the smaller sample volumes from mice did not permit accurate estimation of this highly reactive acrolein since most of the sample was lost during steps involved in generation of stable acrolein metabolites (data not shown). As a reliable surrogate of inhaled SHS, we exposed cell free PBS to SHS under conditions similar to that of both in vitro and in vivo laboratory exposures, as previously reported [33]. Exposure samples were immediately derivatized to generate stable analogues that would permit storage and chromatography analysis at a later time. As seen in Fig. 4, in samples exposed to SHS acrolein concentration was higher than matched air controls (SHS 3935 ± 909.1 ng/mL vs air control 105.70 ± 25.5 ng/mL, *p* < 0.05). Thus, these data demonstrate that acrolein content in SHS may be a significant contributor to harmful effects on airway epithelial ion transport.

### N-acetylcysteine (NAC) protects against SHS -induced CFTR dysfunction in vitro and in vivo

Antioxidants are capable of scavenging reactive aldehydes such as acrolein and thus, prevent its direct interaction with proteins and nucleic acids [39, 40]. To verify the feasibility of overcoming biologic effects of SHS, unbound and reactive acrolein from SHS exposure was analyzed in PBS in presence of NAC, an antioxidant previously shown to competitively bind acrolein faster than other biological molecules/proteins and also proven to protect CFTR function against smoking-related redox stress [4]. Presence of NAC reduced free acrolein in SHS samples to levels approaching what was found in control samples (SHS + NAC 121.50 ± 17.61 ng/mL vs air control 105.70 ± 25.50 ng/mL, *p* < 0.05; Fig. 4). Next, we evaluated the potential of antioxidants to protect CFTR activity from SHS exposure. HBE cells cultured at air liquid interface until differentiation into ciliated epithelium were pre-treated for 30 min with 300uM of NAC, a concentration at which free acrolein was near completely eliminated (Fig. 4), followed by exposure to either control room air or SHS. Following incubation for an hour after SHS exposure, HBE cells were mounted in Ussing Chambers and CFTR activity was analyzed.

**Fig. 5:**
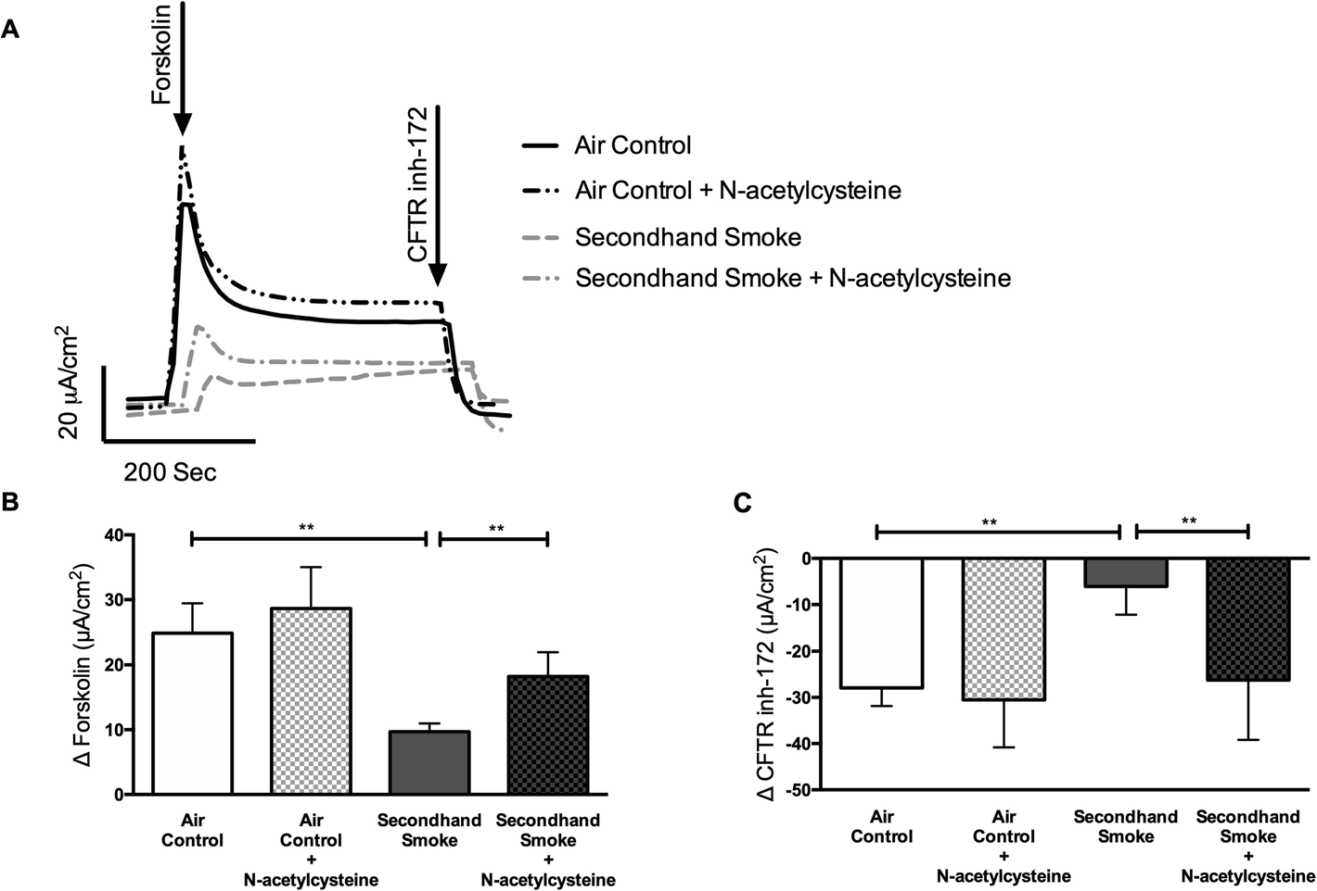
Antioxidants protect against reduced CFTR function by secondhand smoke (SHS) in vitro. **a**. Representative Ussing chamber electrophysiologic tracing of primary human bronchial epithelial (HBE) cells pretreated with N-acetylcysteine (NAC, 300 μM) for 30 min and exposed to either control room air or SHS for 10 min at room temperature. **b**. Summary graphs illustrates changes in forskolin (10 μM)-stimulated CFTR activity in HBE cells that was also evident in Isc change following addition of CFTR specific inhibitor, CFTR_Inh_172 (10 μM, (**c**). *n* = 6–8, *P < 0.05; **P < 0.005

As shown in Fig. 5, compared to values obtained in SHS-exposed HBE cells, pre-treatment with NAC resulted in an 88% protection against CFTR dysfunction (Δ forskolin in μA/cm^2^, SHS + NAC 18.23 ± 3.73 vs SHS 9.70 ± 1.26, *p* < 0.005). Despite SHS-exposure, NAC-treated cells exhibited CFTR activity comparable to control HBE cells exposed to room air (Δ forskolin Isc in μA/cm^2^, SHS + NAC 18.23 ± 3.73 vs air control 24.91 ± 4.53, NS). Trends in chloride ion transport were also verified in response to CFTR specific inhibitor CFTRinh-172 (Fig. 5 c). HBE cells pretreated with NAC retained 62% of forskolin-stimulated CFTR activity when compared to cells affected by SHS (Δ CFTRinh_172_ Isc in μA/cm^2^, SHS + NAC -26.23 ± 12.94 to SHS -6.08 ± 6.12, *p* = 0.005). To verify the benefits of NAC treatment observed in primary HBE cells was not unique to the donor tested, we repeated these studies in immortalized 16-HBE cells known to exhibit more consistent CFTR expression in laboratory cultures. 16-HBE cells exposed to SHS caused a 53% decrease in CFTR function (Supplementary Figure 1 A, B) as compared to control room air (SHS 9.46 ± 3.26 μA/cm^2^ vs air control 20.20 ± 3.35 μA/cm^2^, *p* < 0.05). NAC treatment generated a 95% protection of SHS-induced CFTR loss (SHS 9.46 ± 3.26 μA/cm^2^ vs SHS + NAC 18.43 ± 2.34 μA/cm^2^ in Δ forskolin Isc, *p* < 0.05). Moreover, NAC administration in untreated16HBE or primary HBE cells caused no negative effects on transepithelial electrical resistance and CFTR function in untreated cells. Thus, these data demonstrate the potential of antioxidant therapy to preserve transepithelial chloride transport negatively impacted by SHS exposure without any untoward effects on HBE cell viability and function.

**Fig. 6:**
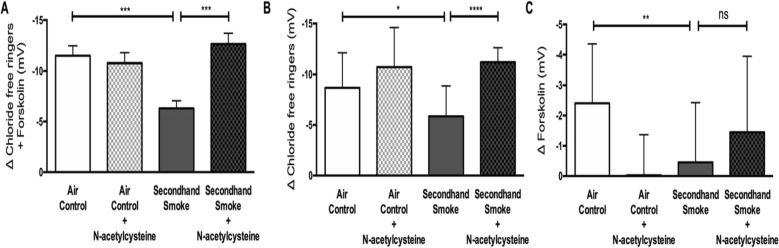
Oral administration of N-acetylcysteine (NAC) prevents loss of CFTR-mediated ion transport in mice exposed to secondhand smoke (SHS)*.***a**. Representative nasal potential difference (NPD) tracing for CFTR-dependent epithelial ion transport in mice that were exposed to either control room air or SHS for 6 weeks with or without NAC added to drinking water (40 mM). b. Summary graphs illustrate changes in total CFTR-dependent voltage following infusion of chloride-free ringers and forskolin (10 μM) across nasal epithelium. Reductions in CFTR activity are separately described for the fraction of channels that were open at baseline and responded to chloride-free ringers (**c**) and the closed fraction that were activated by addition of forskolin (**d**). *n* = 7–19, *P < 0.05, **P < 0.005

To test the protective effects of NAC in vivo, another cohort of A/J mice were exposed to control air or SHS for 5 weeks with half of each group receiving NAC in drinking water. Previous work indicates, oral NAC added to drinking water is readily absorbed in mice and reaches therapeutic levels in blood [27]. After 5 weeks of SHS exposure with and without NAC treatment, in vivo CFTR function was evaluated by NPD. NAC treatment did not alter the total CFTR dependent voltage in control mice exposed to room air (Fig. 6a). As such, all mice handled NAC treatment with no obvious signs of discomfort or loss of body weight (data not shown). When compared to control mice, SHS exposure reduced total CFTR activity by 45% (Δ chloride free ringers + forskolin, SHS − 6.31 ± 3.52 mV vs air control − 11.48 ± 3.65 mV, *p* < 0.0005). Interestingly, NAC treatment fully protected against CFTR dysfunction by SHS (Δ chloride free ringers + forskolin, SHS − 6.31 ± 3.52 mV vs SHS + NAC − 12.63 ± 2.82 mV, *p* < 0.0005). Moreover, with NAC treatment SHS-exposed mice retained CFTR activity that was comparable to control mice (Δ chloride free ringers + forskolin, SHS + NAC − 12.63 ± 2.82 mV vs air control − 11.48 ± 3.65 mV, NS) suggesting maximal benefit of antioxidant therapy against SHS effects on airways (Fig. 6a). Then, we evaluated if NAC administration exerted differential effects on open/closed portions of CFTR channels. As represented in Fig. 6b, portion of CFTR channels that were in open configuration was reduced 32% by SHS (Δ chloride free ringers, SHS − 5.85 ± 3.02 mV vs air control − 8.68 ± 3.43 mV, *p* < 0.05), NAC treatment fully protected the activity of open portion of CFTR channels against SHS exposure (Δ chloride free ringers, SHS + NAC − 11.18 ± 1.42 mV vs air control − 8.68 ± 3.43 mV, NS). Thus, NAC treatment represents a 91% protection from SHS in CFTR channels that are in open configuration (Δ chloride free ringers, air control − 8.68 ± 3.43 mV, SHS − 5.85 ± 3.02 mV and SHS + NAC − 11.18 ± 1.42 mV, *p* < 0.0005). As shown in Fig. 6c, SHS diminished activity of channels in closed state by 81% as compared to air controls (Δ forskolin, SHS − 0.45 ± 1.98 mV vs air control − 2.41 ± 1.95 mV, *p* < 0.005); however, the proportion of closed CFTR channels was only partially modified by NAC (Δ forskolin, SHS − 0.45 ± 1.98 mV vs SHS + NAC − 1.45 ± 2.50 mV, *p* = 0.53).

In addition to ion transport assay in nasal epithelium by NPD, we sought to confirm changes in CFTR function directly in airway explants representative of lungs.

**Fig. 7:**
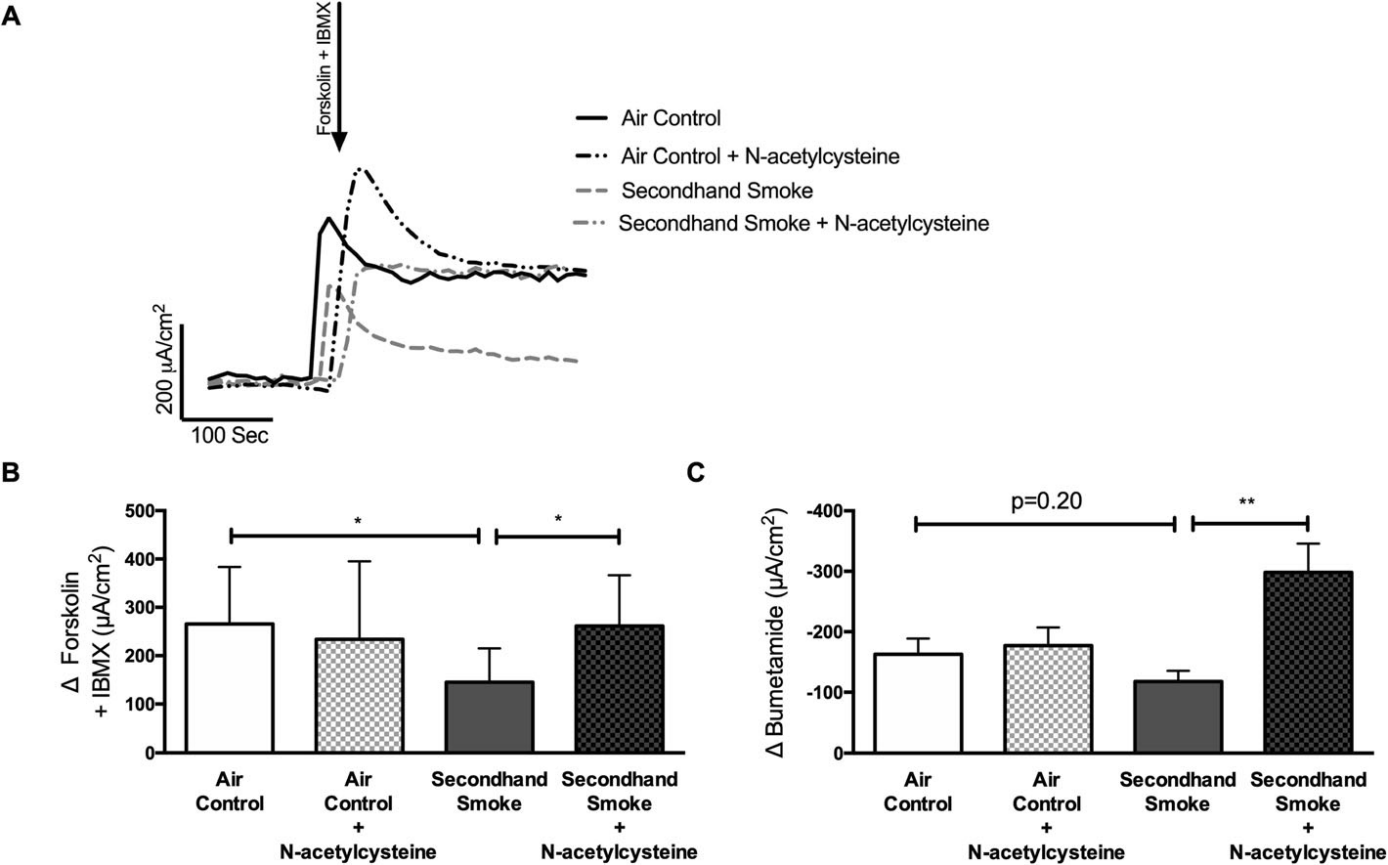
Pretreatment with N-acetylcysteine (NAC) protects against CFTR dysfunction by secondhand smoke (SHS) in murine airways. **a**. Representative Ussing chamber electrophysiologic tracing of excised trachea from mice exposed to either control room air or SHS for 6 weeks in nose-only inhalation system with or without NAC added to drinking water (40 mM). **b**. Summary graph illustrating CFTR activity following addition of forskolin (10 μM) + IBMX (100 μM) under an apical chloride ion gradient generation and presence of ENaC inhibitor amiloride. Subsequent bumetanide (10uM) inhibition of basolateral NKCC1, that generates chloride ions for CFTR channels, was used as a secondary confirmation of CFTR activity (**c**). *n* = 8–10, *P < 0.05

As described in the representative tracing in Fig. 7a, excised murine tracheas were mounted in Ussing chambers to measure CFTR-mediated ion transport activity. When compared to control mice, SHS exposure reduced tracheal CFTR activity by 45% (Δ forskolin + IBMX Isc, SHS 145.40 ± 69.85 μA/cm^2^ vs air control 265.40 ± 118.10 μA/cm^2^, *p* < 0.05). This loss of airway CFTR function by SHS was significantly averted when mice were co-treated with NAC (Δ forskolin + IBMX Isc, SHS 145.40 ± 69.85 μA/cm^2^, SHS + NAC 261.40 ± 105.20 μA/cm^2^, *p* < 0.05). These data validate previously shown benefits of NAC treatment in smoking-induced CFTR dysfunction in human cells [4]. Similar to Fig. 3c, to evaluate antioxidant benefits on chloride-specific ion transport bumetanide, an inhibitor of the Na^+^/K^+^/2Cl^−^ cotransporter, was also tested (Fig. 7c). Compared to controls, there was a trend towards 28% reduction in bumetanide-inhabitable ion transport in SHS-exposed mice (Δ bumetanide Isc, SHS -117.70 ± 52.89 μA/cm^2^ vs air control −162.81 ± 82.48 μA/cm^2^, *p* = 0.20). However, trachea from SHS-exposed mice treated with NAC exhibited a significant increase in bumetanide response as compared to SHS mice (Δ bumetanide Isc, SHS -117.70 ± 52.89 μA/cm^2^ vs SHS + NAC -298.0 ± 83.07 μA/cm^2^; *p* < 0.005) suggesting maximal protection matching that of control mice (Δ bumetanide Isc, SHS + NAC -298.0 ± 83.07 μA/cm^2^ vs air control − 147.50 ± 55.26 μA/cm^2^; NS). Finally, to examine the potential adverse effects of NAC, control mice were also treated with NAC. Air control mice treated with NAC showed no significant difference in stimulated CFTR response (Δ forskolin + IBMX Isc, air control + NAC 234.00 ± 161.40 μA/cm^2^ vs air control 265.40 ± 118.10 μA/cm^2^, NS) or, following inhibition with bumetanide (Δ bumetanide Isc, air control + NAC -177.30 ± 51.42 μA/cm^2^ vs air control − 162.81 ± 82.48 μA/cm^2^, NS). Overall, both in vitro and in vivo electrophysiological data strongly support the administration of antioxidants to protect CFTR function from harmful effects of SHS.

### Effects of CFTR dysfunction by secondhand smoke on airway microanatomy

**Fig. 8:**
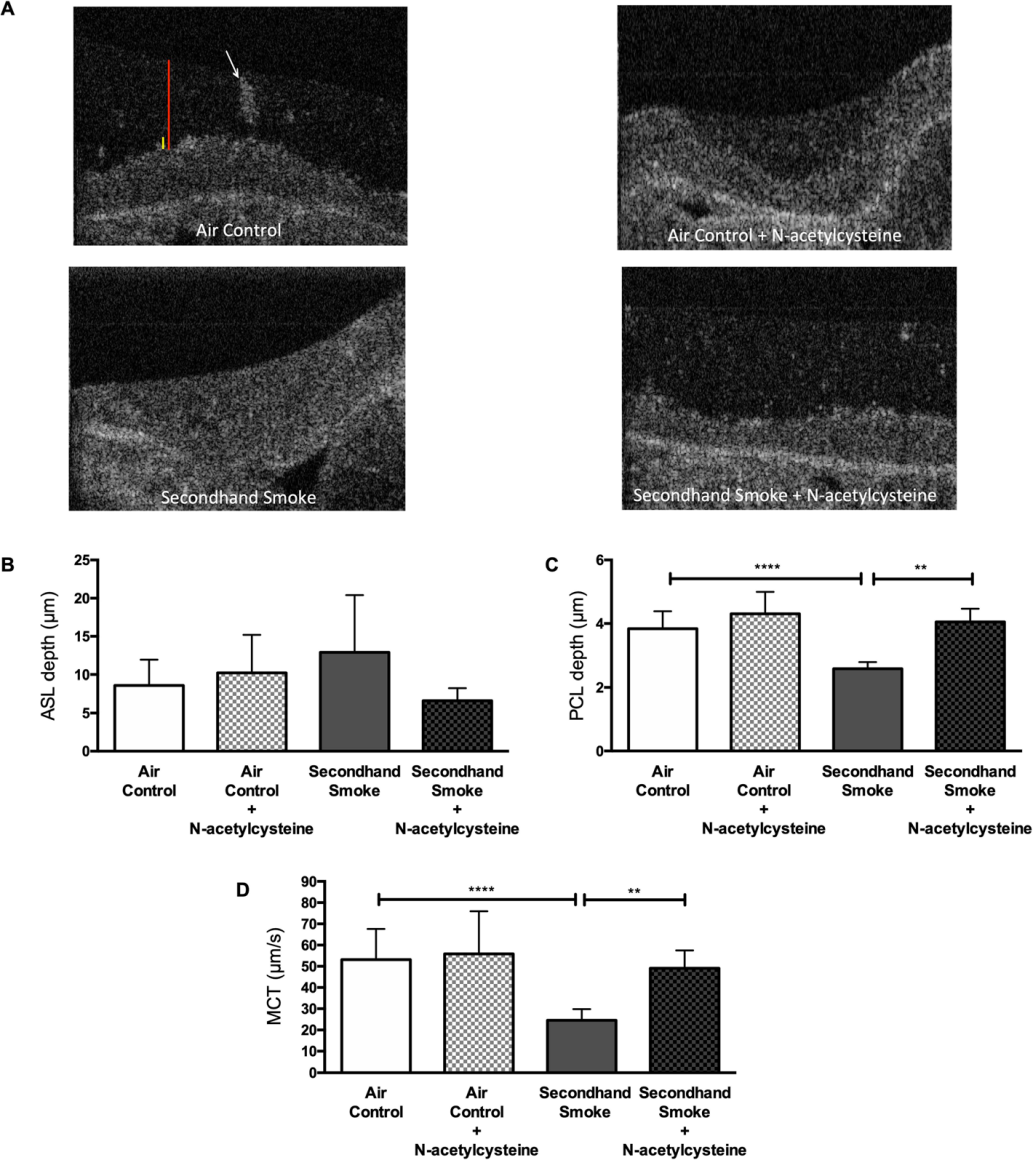
N-acetylcysteine (NAC) prevents secondhand smoke (SHS) effects on airway surface hydration and mucociliary transport (MCT) in mice. **a**. Representative μOCT images of tracheae excised from mice exposed to control room air or SHS for 6 weeks either with or without NAC added to drinking water (40 mM) are shown. Periciliary liquid (PCL) layer and air surface liquid (ASL) depths are indicated by yellow and red bars, respectively. A mucus aggregate that is large and easily noticeable is also shown by a white arrow. It is the movement of such native particles that is used to discern the rate of mucociliary transport (MCT). Summary graphs illustrate total changes in ASL depth (**b**), PCL depth (**c**) and MCT rate (**d**) in excised trachea from each mouse in the study. *n* = 7–19, *P < 0.05, **P < 0.005

Based on the known physiological impact of reduced CFTR function, we next evaluated how SHS exposure alters airway surface properties that are found to be defective in COPD and other airway diseases [41]. To this end, we employed μOCT imaging that allows integrated assessment of airway epithelial cells functions without requiring the use of external contrast dyes or fluorescent microparticles [42]. At the end of 5 weeks of either SHS or control air exposure of mice, freshly excised tracheal segments were imaged by μOCT just before ion transport measurement with Ussing chamber electrophysiology. As shown in the representative images in Fig. 8a, depth of ASL and PCL can be simultaneously estimated in images acquired by μOCT. Based on image analyses made in tissues collected from 3 independent cohorts of mice, SHS exposure was found to cause a partial increase in ASL depth that was statistically insignificant (SHS 12.91 ± 7.51 μm vs air control 8.61 ± 3.34 μm, NS). However, consistent with reduced CFTR function SHS exposure caused a 33% decrease in PCL depth as compared to control room air (SHS 2.58 ± 0.21 μm vs air control 3.84 ± 0.55 μm, *p* < 0.0005). When time-lapsed μOCT videos are analyzed, the movement of native mucus particles appears as streaking. Based on the angle of these streaks the rate of mucus transport on the airway surface can be estimated (larger angles correlate with greater MCT), as previously reported [32]. As summarized in Fig. 8d, compared to air controls SHS exposure reduced MCT by 54% (SHS 24.54 ± 5.25μmWe/s vs air control 53.17 ± 14.48 μm/s, *p* < 0.00005). Collectively, these data indicate that reduced CFTR-mediated anion secretion by SHS exposure results in inefficient hydration of airway mucosal surface causing severely impaired MCC in mice.

### Reversal of SHS-induced CFTR dysfunction by N-acetylcysteine (NAC) improves airway surface hydration and mucus transport in vivo

To test whether rescue of CFTR function by antioxidant NAC could also augment airway surface hydration and MCC, we again relied on μOCT imaging of tracheal explants. In both groups of mice, the influence of NAC treatment on ASL depth was modest and statistically insignificant (Fig. 8b). However, administration of NAC in SHS-treated mice fully prevented depletion of PCL depth by SHS (SHS 2.58 ± 0.21 μm vs SHS + NAC 4.05 ± 0.41 μm, *p* < 0.005). With NAC treatment, PCL depth in SHS-exposed mice remained at levels comparable to that of air control mice (Air Control 3.84 ± 0.55 μm). More importantly, as shown in Fig. 8d, NAC treatment significantly protected SHS exposed mice from reduced MCT rate (SHS 24.54 ± 5.25 μm/s vs SHS + NAC 49.07 ± 8.46 μm/s, *p* < 0.005). As such, administration of NAC did not further increase MCT rates in control mice indicating physiologic ceiling for MCT (air control + NAC 55.86 ± 20.09 μm/s vs air control 53.17 ± 14.48 μm/s, NS). Representative videos for each treatment group are shown in Supplementary figures 2-5. The in vivo data presented here illustrate the potential of antioxidant therapy to address the pathologic consequence of reduced CFTR-mediated ion transport on airway surface hydration and MCT rates.

**Fig. 9:**
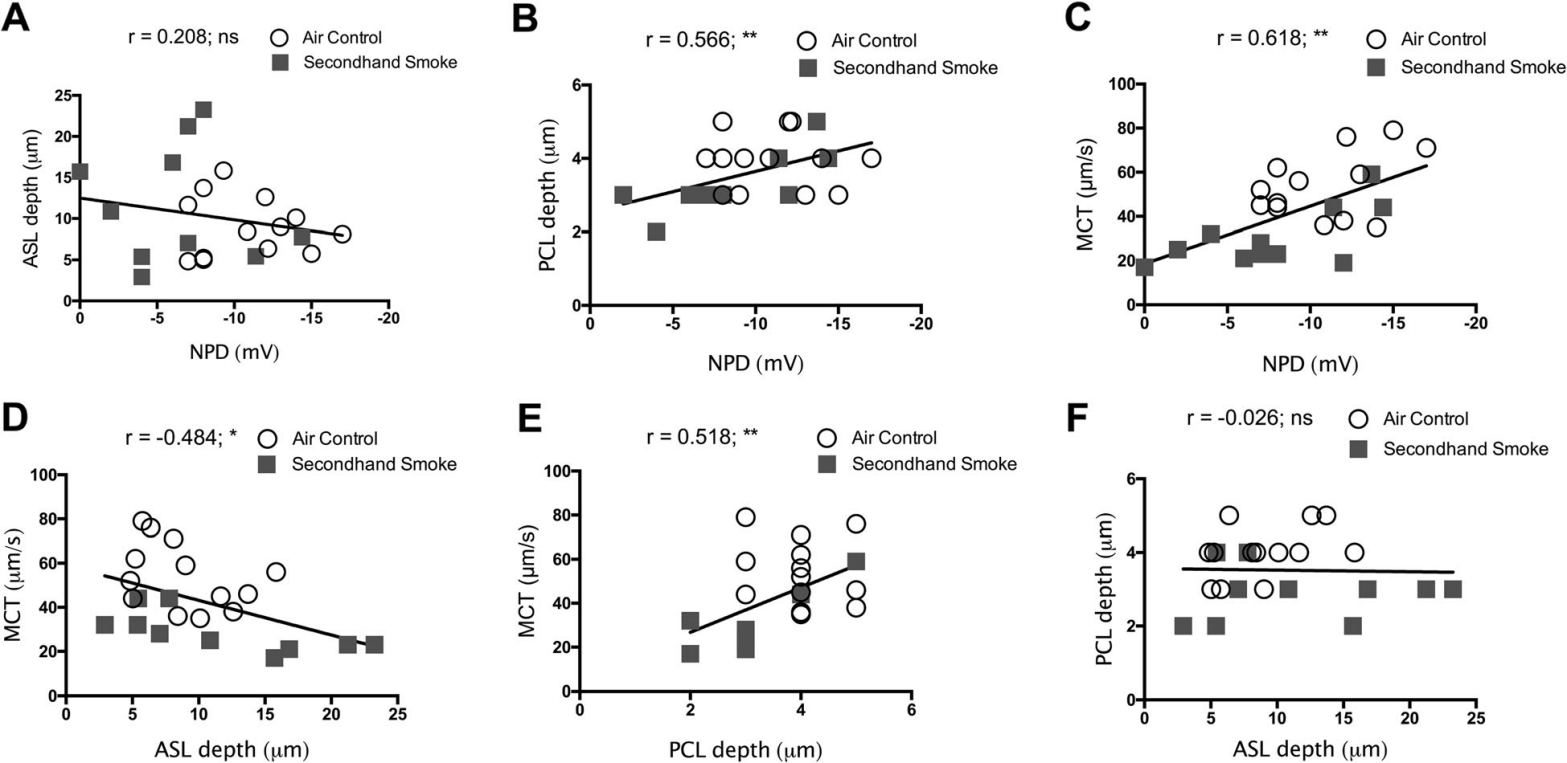
Correlation of secondhand smoke (SHS)-induced CFTR dysfunction to altered functional microanatomy of murine airways. Functional relationships between CFTR activity by nasal potential difference (NPD) and data obtained from μOCT image analyses regarding periciliary liquid (PCL) depth, air surface liquid (ASL) depth, and mucociliary transport (MCT) on trachea were analyzed by Pearson correlations using SPSS program. Matched data from experiments between 23 mice exposed room air (open circles) and SHS (grey squares) were included. All correlations were two tailed, 95% confidence, with confidence interval expressed lower bound and upper bound; *P = < 0.05, **P = < 0.005. **a.** Correlation between NPD and ASL depth had a weak positive association (*r* = 0.208) that was statistically insignificant (*p* = 0.340) **b.** Correlation between NPD and PCL depth demonstrated a moderate positive association (*r* = 0.566) that was significant (**p = < 0.005). **C.** Linear curve fits for NPD and MCT indicated a moderate positive association (r = 0.618) that was significant (**p = < 0.005). **d.** Examining MCT relationship with ASL depth revealed a moderate negative association (*r* = − 0.484). that was significant (*p = < 0.05). **e.** Correlation of MCT with PCL depth achieved a significant (*p = < 0.05) but, moderate positive association (*r* = 0.518). **f.** Association analysis between ASL and PCL depths revealed a weak negative association (*r* = − 0.026, *p* = 0.905)

Next, we attempted to generate functional interrelationships between CFTR activity and functional parameters of the airway surface that were simultaneously determined by μOCT image analysis, a unique advantage of this technology. Our data from murine trachea from both control as well as smoke-exposed mice demonstrate in vivo CFTR activity was directly related to the status of PCL hydration (*r* = 0.566, **p = < 0.005; Fig. 9b) and rate of MCT (*r* = 0.618, **p = < 0.005; Fig. 9c). Consistent with recent observations made in CFTR knockout rat airways [31], PCL depth was also significantly associated with MCT rate (*r* = 0.518, **p = < 0.005; Fig. 9e), validating the importance of PCL hydration by optimum CFTR activity towards the maintenance of physiologic MCC. However, despite ASL depth being inversely related to MCT rate (*r* = − 0.484, *p = < 0.05; Fig. 9d), the association was not evident for ASL depth and NPD (r = 0.208, NS; Fig. 9a) or, ASL depth and PCL depth (*r* = − 0.026, NS; Fig. 9f) suggesting factors beyond altered CFTR activity such as increased mucus secretion/increased accumulation by SHS may also negatively impact MCC [43]. Thus, based on our data SHS-induced CFTR dysfunction directly results in reduced PCL depth causing diminished MCT rate in SHS-exposed murine airways.

## Discussion

Exposure to secondhand smoke (SHS) is associated with a number of respiratory diseases [44]. Although evidence is unequivocal that avoiding exposure to inhaled irritants like SHS is critical to limit lung diseases, a large number of individuals including vulnerable children continue to be exposed to SHS around the world. Thus, this preventable cause of the illness remains a major health concern in spite of improving regulations limiting to SHS exposure in public spaces [14]. Yet, there are knowledge gaps regarding molecular understanding of SHS effects on lung physiology.

For example, reduced lung defense via delayed mucociliary clearance has been suggested to result in increased chronic bronchitis among never smokers exposed to SHS [22, 23].

Previous in vitro studies have demonstrated that exposure to SHS results in defective CFTR-mediated anion transport, and is likely to contribute to increased respiratory manifestations among passive smokers [21]. Using many complementary studies that include the first evaluation of the effects of SHS on CFTR function in an isogenic animal model, we demonstrate that SHS exposure is causally linked to CFTR dysfunction in vivo. In addition, in vitro and in vivo experiments demonstrate that reactive aldehydes such as acrolein present in cigarette smoke may be directly affecting CFTR activity and can be pharmacologically protected by antioxidants such as NAC that neutralize free acrolein.

It is widely accepted that when reduced CFTR-mediated chloride ion transport is unable to support optimal hydration of airway surface fluid layers, MCC rate is reduced and unwanted particulate debris, pathogens, and toxins accumulate in lung [45, 46]. However, it was not known whether SHS effects on CFTR ion transport can cause abnormalities in mucus properties or, they can be compensated by other physiologic mechanisms. Here, using a novel functional anatomic imaging we also demonstrate the deleterious effects of SHS on CFTR dysfunction cause reduced hydration of airway surface liquid, PCL, that lubricate ciliary beating, providing direct evidence that SHS exposure contributes to loss of effective mucus transport. Further, recovery of CFTR function by NAC treatment restored the depth of PCL layer and physiologic rates of mucus clearance in mice exposed to SHS. Despite individuals that are heterozygous for CFTR mutations exhibit only a partial reduction in CFTR function, they remain more susceptible to respiratory infections than the general population. This implies CFTR dysfunction by SHS may also account for increased infectious burden among passive smokers [47]. Consistent with this notion, Ni et al. have reported that SHS significantly reduces phagocytic clearance of bacteria by depleting membrane expression of CFTR in macrophages extending the pathologic significance of CFTR dysfunction beyond the airway epithelium [48].

Previous genotype-phenotype correlations for CFTR mutations have established that even modest reductions in CFTR function increase the risk of several pulmonary and extra-pulmonary diseases [4, 49]. Thus, CFTR decrements observed in our SHS studies may be highly relevant to the pathogenesis of airways diseases with decreased MCC including, COPD-related chronic bronchitis, a major COPD phenotype that we have previously shown to be causally associated with acquired defects in CFTR expression and function [4, 9] Moreover, the recent emergence of small molecule drugs such as Ivacaftor that can increase CFTR activity, including in patients with normal CFTR genetics, has opened new avenues of potential treatments for respiratory illnesses like COPD -related chronic bronchitis [50].

Acrolein is a highly reactive aldehyde, which is present in higher concentrations in SHS than traditional mainstream smoke that flows through filtered tips. Given the abundance of acrolein in SHS and its ability to reduce the probability of opening CFTR channels, a pharmacological reversal of CFTR dysfunction could be an important therapeutic approach to address the pathological manifestations of SHS. Based on the effective neutralization of free acrolein by antioxidant NAC, we evaluated antioxidant therapy in vivo and found it to be a viable approach to limit smoking-induced CFTR defects. While beneficial effects of NAC observed on lung function and exacerbations in COPD patients have largely been attributed to its antioxidant and mucolytic properties and the significance of acrolein neutralization and changes in CFTR function in these patients has not been studied [51, 52].

Several different mechanisms are known to mediate acquired CFTR dysfunction by cigarette smoke. Evidence from multiple groups including us reported the involvement of oxidative stress in CFTR dysfunction [53]. Thus, while our data specifically addresses the role of acrolein in CFTR dysfunction, these data do not exclude the role of other oxidative agents present in smoke [53] such as heavy metal toxins like cadmium [11], hypoxic injury [54] and nicotine [55, 56]. In contrast, Savitsky and colleagues found that the particular matter in SHS was mostly responsible for CFTR dysfunction. As discussed here, there is growing evidence that volatile and non-particulate content in smoke such as acrolein and nicotine are sufficient to reduce CFTR function in vivo*.* Besides, protection of epithelial ion transport function by NAC in mice exposed to SHS establishes the relative importance of acrolein and other oxidants in SHS in mediating CFTR dysfunction. These data are also in line with our previous reports that acrolein confers abnormal CFTR gating by inducing post-translational modifications that alter its functional properties [57] in airways [58] as well as in other extra-pulmonary tissues causing systemic CFTR function [4].

The antioxidant NAC is known to mitigate the biologic effects of oxidative reactants like acrolein via direct and indirect mechanisms [59, 60]. The direct approach involves thiol groups in NAC interacting with electrophilic groups on oxidants [59, 61] whereas the indirect mechanism involves NAC functioning as a precursor for glutathione (GSH), a major physiologic antioxidant [62] that is primarily synthesized in liver and operates throughout the body to reduce oxidant injury. Data presented here confirm NAC’s ability to successfully scavenge acrolein and prevent disruption of CFTR mediated chloride transport in vitro and in SHS-exposed mice. To our knowledge, this is the first in vivo evidence of using an antioxidant to treat SHS-induced CFTR dysfunction. However, the indirect mechanisms involving increase GSH reserves might cause additional benefits beyond CFTR dysfunction.

Our results corroborate previous works including Moldeus et al. which demonstrated NAC’s ability to mitigate the toxic effects of tobacco smoke condensate in human bronchial fibroblast cells [59]. However, Moldeus et al. concluded NAC’s ability to scavenge toxic tobacco smoke chemicals in vivo would be ineffective due to low NAC bioavailability from being metabolized to a glutathione metabolite. Similar observations were made to explain the inconsistent benefits observed with NAC between COPD studies. However, as reported recently, higher doses of NAC achieved higher tissue levels of glutathione and offered clinically-meaningful improvements in disease progression [63, 64]. However, these studies weren’t limited to passive smokers only nor directly assess changes in acrolein levels or CFTR activity. In addition, our data indicate the overall benefits of NAC treatment on promoting epithelial ion transport may involve ion channels beyond CFTR. Bumetanide-sensitive changes in Isc represent the function of basolateral Na-K-Cl (NKCC) cotransporters. As such these transporters serve to supply chloride ions for CFTR channels on the surface epithelium. Interestingly, protection of CFTR function by NAC in SHS exposed mice coincided with increased bumetanide-sensitive changes in Isc. Since there is an abundance of literature demonstrating NAC direct effects on mucus viscosity, we haven’t directly addressed that in this report. Thus, the improvements we observed in MCC in SHS-exposed mice following NAC administration may also involve direct modification of mucus viscosity in addition to enhanced hydration of PCL and mucus layers.

Overall, a major strength of this study is the consistency observed in in vitro, in vivo, and ex vivo assessments of CFTR-mediated ion transport. Percentage differences in CFTR activity due to SHS exposure remained comparable across all model systems. Furthermore, our results are strengthened through the μOCT observations demonstrating NAC’s protection of CFTR mediated chloride ion transport translates to the preservation of physiologic PCL depth and MCC in mice exposed to SHS. Major limitations of the current study include assessment of SHS at a single dose that may not represent the observations across all passive smokers. In addition, reflecting the short duration of the study mice exhibiting CFTR dysfunction do not recapitulate overt bronchitis reminiscent of COPD. Thus, future studies may only clinically-relevant dose-response assessments for SHS on CFTR function. Moreover, our data do not directly assess if NAC treatment might benefit MCC rates in COPD patients who have either quit smoking or avoid exposure to SHS. It would also be interesting if NAC treatment might improve MCC defects in electronic cigarette users or those passively exposed to vapors from electronic cigarettes.

## Conclusion

In summary, data presented here conclude secondhand smoke exposure causes CFTR dysfunction and adversely impacts physiologic airway surface hydration and mucociliary clearance in vivo. Besides, these data implicate a causal role for reactive aldehydes such as acrolein in mediating CFTR defects in mice exposed to SHS. Further, using an antioxidant we also demonstrate a viable approach to overcome cigarette smoke effects on CFTR function and mucus transport rate in patients with muco-obstructive airways disease. These results improve our understanding of the deleterious effects of SHS on lung health and explain the role of acquired CFTR dysfunction in COPD pathogenesis among passive smokers.

## Supplementary information

**Additional file 1. Supplement Figure 1.** Antioxidants protect against reduced CFTR function by secondhand smoke (SHS) in 16HBE cells. **A.** Summary graph illustrates changes in forskolin (10 μM)-stimulated CFTR activity in 16HBE cells exposed to either 10 min of SHS or control room air. Changes in CFTR function when pretreated with N-acetylcysteine (NAC, 300 μM) for 30 min before SHS exposure are shown. *n* = 4–6, *P < 0.05.

**Additional file 2. Supplementary Video 1.** Air Control Video.

**Additional file 3. Supplementary Video 2.** Air Control + N-acetylcysteine Video.

**Additional file 4. Supplementary Video 3.** Secondhand Smoke Video.

**Additional file 5. Supplementary Video 4.** Secondhand Smoke + N-acetylcysteine Video.

## Data Availability

The datasets used and/or analyzed during the current study are available from the corresponding author on request.

## Abbreviations

ALI: Air-liquid interface
ASL: Air surface liquid
ATP: Adenosine triphosphate
cAMP: cyclic adenosine monophosphate
CF: Cystic fibrosis
CFTR: Cystic fibrosis transmembrane conductance regulator
CFTRinh-172: CFTR inhibitor number 172
Cl^-^: Chloride ions
COPD: Chronic obstructive pulmonary disease
DMSO: Dimethyl sulfoxide
ENaC: Epithelium sodium ion channel
HBE: Human bronchial epithelial
IACUC: Institutional Animal Care and Use Committee
Isc: Short circuit current
MCC: Mucociliary clearance
MCT: Mucociliary transport
MS: Mainstream smoke
NAC: N-acetylcysteine
NKCC: Na^+^-K^+^-Cl^−^ cotransporter
NPD: Nasal potential difference
PCL: Periciliary layer
SD: Standard deviation
SHS: Secondhand smoke
UAB: University of Alabama at Birmingham
WT: Wild type
μOCT: micro optical coherence tomography
