# Supplementary material for: Evaluation of secondhand smoke effects on CFTR function in vivo
Source: Respir Res. 2020 Mar 20;21:70. doi: 10.1186/s12931-020-1324-3 (PMC7082971; doi:10.1186/s12931-020-1324-3)
Supplement: Supplementary file 1 — Additional file 1. Supplement Figure 1. Antioxidants protect against reduced CFTR function by secondhand smoke (SHS) in 16HBE cells. A. Summary graph illustrates changes in forskolin (10 μM)-stimulated CFTR activity in 16HBE cells exposed to either 10 min of SHS or control room air. Changes in CFTR function when pretreated with N-acetylcysteine (NAC, 300 μM) for 30 min before SHS exposure are shown. n = 4–6, *P < 0.05. [file 12931_2020_1324_MOESM1_ESM.docx]

**Supplement Figure 1:** Antioxidants protect against reduced CFTR function by secondhand smoke (SHS) in 16HBE cells. **A.** Summary graph illustrates changes in forskolin (10 μM)-stimulated CFTR activity in 16HBE cells exposed to either 10 minutes of SHS or control room air. Changes in CFTR function when pretreated with N-acetylcysteine (NAC, 300 μM) for 30 min before SHS exposure are shown. n = 4-6, *P<0.05.

**Supplement Figure 1**
